# Twenty-six years of misdiagnosis as gastroesophageal reflux disease in a patient with achalasia presenting with chronic dysphagia: a case report

**DOI:** 10.3389/fmed.2026.1994033

**Published:** 2026-10-26

**Authors:** Yanping Dai, Yang Liu, Jun Ye

**Affiliations:** 1 Department of Gastroenterology, Chonggang General Hospital, Chongqing, China; 2 Department of Gastroenterology, The First Affiliated Hospital (Southwest Hospital) of Army Medical University (Third Military Medical University), Chongqing, China

**Keywords:** achalasia, case report, dysphagia, misdiagnosis, peroral endoscopic myotomy (POEM)

## Abstract

Impaired relaxation of the lower esophageal sphincter and absent peristalsis throughout the esophageal body constitute the core pathological manifestations of achalasia. High-resolution manometry is rarely accessible at primary medical institutions. Coupled with substantial overlap between its clinical manifestations and gastroesophageal reflux disease, missed diagnosis and prolonged misdiagnosis occur frequently. This report describes a 70-year-old male patient afflicted with progressive dysphagia for as long as 26 years. Repeated esophagogastroduodenoscopy (EGD) misclassified his complaint as gastroesophageal reflux disease, and proton pump inhibitors only relieved symptoms transiently. His clinical status deteriorated drastically over the past three months, accompanied by a 5-kilogram weight loss. Combining typical clinical history, chest computed tomography, EGD and the pathognomonic bird-beak deformity observed on upper gastrointestinal barium swallow, a clinical diagnosis of achalasia was established. His initial Eckardt score was 8. The patient received peroral endoscopic myotomy (POEM) without intraoperative complications, and his dysphagia resolved rapidly after surgery. No symptom relapse was recorded in the 6-month postoperative follow-up. His body weight recovered by 4 kg, and the follow-up Eckardt score dropped to 0. This case delivers critical clinical implications. Clinicians tend to confuse achalasia with gastroesophageal reflux disease, and short-term symptomatic improvement induced by proton pump inhibitors may potentially mask the underlying disease and contribute to diagnostic delay. When high-resolution manometry cannot be performed, a definitive diagnosis can be reached through typical medical history, barium radiography and EGD evaluation. POEM delivers satisfying safety profiles and prominent short-term efficacy for patients suffering from long-standing achalasia.

## Introduction

Achalasia (AC) refers to a primary esophageal motility disorder. Degeneration of inhibitory intermuscular esophageal neurons serves as its core pathological lesion, which subsequently triggers dysfunctional relaxation of the lower esophageal sphincter and complete loss of peristalsis within the esophageal body. Its global annual incidence approximates 0.78 per 100,000 individuals. Clinically, patients typically present with progressive dysphagia, food regurgitation, substernal discomfort and unexplained weight loss (1–4). Regarded as the gold standard for achalasia diagnosis, high-resolution manometry (HRM) enables subtyping under the Chicago classification and guides individualized therapeutic strategies (5). Restricted by equipment expenses and technical thresholds, however, HRM has not been routinely available in most grassroots and prefecture-level hospitals across China, creating a major barrier to early confirmation of diagnosis.

A multicenter cohort study published in 2024 revealed that achalasia patients waited a median of 78.6 months from symptom onset to definitive diagnosis, while 37.3% of affected individuals endured symptoms for more than a decade (6). Case reports documenting misdiagnosis lasting over 20 years remain scarce worldwide. Delayed management gradually induces irreversible esophageal dilatation and malnutrition in some patients, which severely impairs long-term quality of life. Clinical symptoms of achalasia overlap heavily with gastroesophageal reflux disease (GERD). When patients complain of swallowing disturbances, empirical proton pump inhibitor (PPI) therapy is often prescribed without further differentiation. Though PPIs alleviate mucosal inflammation secondary to retained esophageal contents temporarily, they fail to correct fundamental esophageal motility defects. A false therapeutic response, characterized by transient symptom relief followed by aggravated discomfort after drug discontinuation, solidifies the erroneous GERD diagnosis and prolongs the period of misdiagnosis.

Peroral endoscopic myotomy (POEM) is recommended as a first-line minimally invasive intervention for achalasia. Pooled data from meta-analyses document a 94% short-term symptom remission rate, with favorable therapeutic effects observed across different Chicago classification subtypes. Compared with balloon dilatation and laparoscopic Heller myotomy, POEM boasts minimal trauma, accelerated postoperative recovery, and customizable myotomy length, rendering it safe and efficacious for moderate-to-severe cases with extended disease duration (7). Existing clinical investigations mostly enroll patients with relatively short disease courses. Clinical experience addressing patients with more than two decades of untreated achalasia remains limited. Conventional manometry and barium esophagogram are long-validated tools to diagnose this disorder. Even so, fit-for-use standardized diagnostic protocols are hard to come by for grassroots care sites. These local institutions have no access to esophageal manometry technology whatsoever, and that includes high-resolution manometry (HRM), a gap reflected in day-to-day clinical routines.

We hereby report an elderly patient whose achalasia remained misdiagnosed for 26 years. The full clinical journey of repeated misdiagnosis as GERD is systematically summarized. Non-invasive definitive diagnosis was achieved by synthesizing chest computed tomography, EGD findings and the characteristic bird-beak sign on barium swallow. Short-term therapeutic outcomes of POEM in severe long-duration achalasia were assessed via follow-up visits. Combined with recently published domestic and international literature, core contributors to persistent misdiagnosis at primary care facilities were analyzed, and simplified diagnostic protocols for regions lacking HRM access were explored. This case provides evidence-based references and clinical warnings for frontline clinicians to rapidly identify and standardize management of covert achalasia.

## Case presentation

A 70-year-old male was admitted with a chief complaint of progressive dysphagia lasting 26 years and clinical aggravation over the prior three months. He denied previous hypertension, diabetes, surgical trauma, or a family history of digestive malignancies. Dysphagia upon solid food intake first emerged in 2000. Unremarkable endoscopic findings from outside hospitals prompted intermittent oral omeprazole, which yielded fleeting symptom relief. Recurrent and progressively worsening dysphagia plagued the patient over the subsequent 26 years, eventually extending to liquid meals alongside substernal pain. Five EGDs were performed at multiple medical institutions, all yielding diagnoses of GERD, reflux esophagitis and gastric stasis. Long-term oral regimens including PPIs, prokinetic agents and digestive enzymes produced only temporary improvement, and his condition advanced continuously. In February 2026, the patient experienced immediate vomiting after liquid intake and could barely maintain normal oral feeding, accompanied by a 5-kg weight drop within three months. He was therefore hospitalized in the Gastroenterology Department of our institution.

Fasting for 48 h was ordered after admission to complete comprehensive examinations. EGD demonstrated multiple contractile rings starting 30 cm from the incisors, marked dilatation of the upper esophagus with copious retained food and fluid. No ulcerative or space-occupying lesions were visualized after thorough irrigation and suction. The cardia appeared markedly constricted, and significant resistance was encountered during endoscopic passage (Figures 1A–C). Chest computed tomography demonstrated dilatation of the upper and mid-esophagus, thickening of the distal esophageal wall, and intraluminal food retention (Figures 1D,E). Given this elderly patient's weight loss and distal esophageal wall thickening, we rigorously excluded malignancy-related pseudoachalasia. Serial EGDs with complete esophageal lavage detected no neoplastic masses or ulcerations at the cardia or esophagogastric junction. Distal esophageal wall thickening on CT reflected obstruction-induced muscular hypertrophy and fibrosis, with no focal mass, mediastinal lymphadenopathy or infiltrative features. A 26-year indolent clinical course with acute deterioration limited to the prior three-month period contradicted the short disease trajectory typical of malignant pseudoachalasia. Moreover, full symptom resolution and weight gain after POEM further discounted a malignant etiology. Upper gastrointestinal barium radiography identified pathognomonic bird-beak narrowing at the esophagogastric junction, delayed barium transit and dilated proximal esophagus (Figure 1F). Integrating clinical history, EGD and radiological manifestations, a clinical diagnosis of achalasia was made; the radiological esophageal dilatation grade was grade III, which does not correspond to Chicago classification type III, with an Eckardt score of 8.

**Figure 1:**
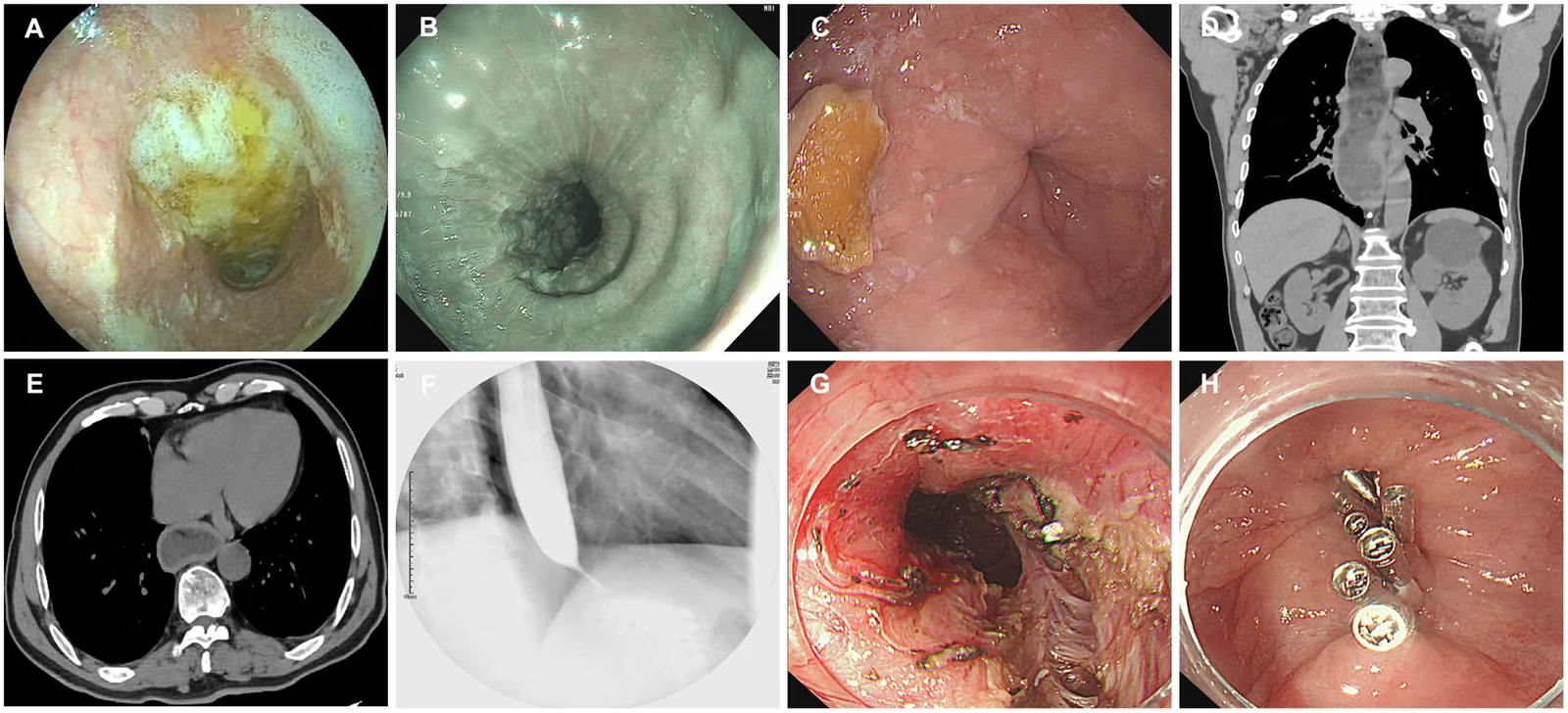
Endoscopic, imaging manifestations and intraoperative findings of the patient with long-course achalasia. **(A–C)** Esophagogastroduodenoscopy (EGD) shows severe esophageal dilatation with massive retained food debris, multiple esophageal contractile rings, and cardia constriction; (D and E) Chest CT demonstrates dilated upper and middle esophagus, thickened lower esophageal wall and intraluminal food retention; **(F)** Upper gastrointestinal barium radiography presents characteristic bird-beak stenosis at the esophagogastric junction with delayed barium emptying; (G and H) Intraoperative endoscopic images obtained during POEM demonstrate complete transection of the submucosal circular muscle bundles, followed by sealing of the mucosal incision using titanium clips.

After diagnosis, multidisciplinary consultation was conducted to evaluate the case, and POEM surgery was determined as the treatment option. Under general anesthesia with endotracheal intubation, a mucosal incision was made on the right posterior esophageal wall approximately 30 cm from the incisor teeth. A 12-cm-long submucosal tunnel was then constructed along this plane. Intraoperative inspection revealed diffuse submucosal and muscular fibrosis resulting from 26 years of chronic esophageal obstruction. Both blunt and sharp dissection were therefore needed to develop the tunnel and perform myotomy. All circular muscle bundles within the tunnel were fully transected; the myotomy extended 2-3 cm across the esophagogastric junction into the gastric cardia (Figure 1G). The mucosal opening was sealed with titanium clips upon completion of the procedure (Figure 1H). No severe intraoperative complications such as perforation or hemorrhage occurred. Dysphagia resolved completely on postoperative day 2, and the patient tolerated oral intake. Discharge was achieved smoothly on postoperative day 3.

Follow-up observation: A telephone follow-up was conducted six months after surgery. No recurrence of dysphagia, regurgitation or substernal pain was documented. The patient tolerated both liquid and solid diets normally, with a 4-kg weight recovery. The repeated Eckardt score fell to 0. Only telephone-based clinical follow-up was performed in this study; follow-up EGD, esophageal pH-impedance monitoring and other objective reflux assessments were not implemented, so subclinical postoperative reflux might be missed. This limitation has been explicitly acknowledged.

## Discussion

This rare case features severe achalasia with a diagnostic delay of 26 years. A clinical diagnosis was established via EGD and radiological examinations without HRM, and POEM yielded favorable short-term clinical outcomes. Drawing upon contemporary clinical research, this discussion elaborates three dimensions: inherent flaws in conventional clinical reasoning, alternative diagnostic workflows for primary care centers, and the adaptability of POEM for patients with pathologically remodeled esophagus from decades-long achalasia. Limitations inherent to this single-case report are also addressed.

Persistent misdiagnosis spanning over two decades stems primarily from rigid diagnostic frameworks centered on reflux disorders, rather than mere symptomatic overlap. Delayed diagnosis of achalasia is partially attributable to limited clinician awareness and inadequate outpatient history-taking. Early achalasia may trigger regurgitation and substernal discomfort that closely imitate GERD-type reflux. As the disease evolves, distinct clinical signs appear: progressive dysphagia, nocturnal cough, reliance on water to clear food, and compensatory postural adjustments like standing or arm-raising during meals to increase intrathoracic pressure for bolus passage. These features differ greatly from classic GERD. Patients often focus on reflux-related symptoms but do not volunteer their compensatory eating patterns. Without deliberate, comprehensive history collection, providers may make a premature GERD diagnosis and overlook important clues of esophageal motility disorders. Pathogenic mechanisms of GERD and achalasia stand in stark opposition. While GERD arises from impaired anti-reflux function of the lower esophageal sphincter, achalasia develops due to depleted inhibitory intramural neurons that abolish sphincter relaxation (8, 9). When patients present with dysphagia, regurgitation and substernal pain, clinicians frequently assign a preliminary GERD diagnosis. Esophageal retention and mild mucosal inflammation visualized on EGD are uniformly attributed to acid-mediated injury. PPIs solely mitigate mucosal irritation induced by stagnant food residues without repairing underlying esophageal motility disorders, and transient symptom relief halts further differential diagnostic workups. Sustained esophageal obstruction triggers progressive complications including muscular fibrosis, luminal dilatation, malnutrition and elevated risks of mucosal dysplasia. These observations urge a tiered diagnostic strategy. For patients who achieve only brief relief from PPIs alongside worsening dysphagia and unintentional weight loss, physicians must abandon reflex GERD assumptions and implement esophageal morphological imaging to avoid missed diagnoses reliant solely on EGD.

For patients with persistent, unexplained dysphagia, non-diagnostic EGD results should not lead clinicians to repeat endoscopic assessments. As seen in our case, five prior EGDs failed to yield a correct diagnosis. If endoscopy cannot account for persistent swallowing complaints, providers ought to turn to alternative tests such as barium radiography or manometry, instead of pursuing repeated endoscopic procedures. Reliable diagnosis hinges on thorough clinical history-taking alongside well-chosen diagnostic tests. Sophisticated imaging cannot make up for superficial symptom assessment, just as detailed history alone cannot substitute for objective examinations. Combined use of these tools helps clinicians differentiate achalasia from gastroesophageal reflux disease among dysphagic patients.

The patient had five prior EGDs performed at outside hospitals over 26 years. Complete original imaging could not be recovered. These examinations took place at geographically separate facilities, most of which lacked consistent digital archiving capabilities two decades ago. Characteristic endoscopic hints, such as esophageal food-fluid retention, luminal dilatation, and a tight cardia on retroflexion, were very likely present on those earlier studies. These markers can be detected by alert endoscopists even for non-advanced achalasia. Nevertheless, without sufficient knowledge of motility disorders, these signs are easily misread as GERD-related gastric stasis or reflux-derived changes, which probably accounts for this patient's repeated diagnostic failure.

HRM enables Chicago subtyping and personalized surgical planning, yet shortages of this equipment at grassroots facilities cannot be resolved in the short term. Developing low-cost, non-invasive combined diagnostic pathways carries vital clinical significance. Reliance on the isolated bird-beak sign from barium swallow delivers limited diagnostic accuracy. Chest computed tomography, EGD and barium radiography function synergistically as an integrated diagnostic chain. Computed tomography quantifies esophageal dilatation and rules out extrinsic mediastinal compression. EGD evacuates luminal debris, excludes malignant cardia strictures, and directly visualizes sphincter constriction. Dynamic barium imaging captures delayed esophageal emptying and conical narrowing at the esophagogastric junction (10). Although this triple-test panel suffices for clinical confirmation, it lacks objective prognostic markers provided by HRM, such as disease subtypes and resting sphincter pressure, constituting an unavoidable shortcoming of primary care diagnostic protocols.

Epidemiological data indicate achalasia patients experience a median diagnostic delay of 78.6 months after symptom onset, with 37.3% suffering symptoms over 10 years prior to definitive diagnosis (6). Combined high-resolution manometry and Endoflip testing can accurately assess esophagogastric junction compliance and peristaltic activity. These technologies permit reassessment of patients once diagnosed with refractory GERD, some of whom are subsequently reclassified as having achalasia or EGJOO. This highlights that underlying EGJ outflow motility disorders ought to be considered for patients with putative GERD exhibiting merely partial or transient improvement under PPI therapy.

International clinical guidelines endorse POEM as the primary minimally invasive procedure for achalasia. Meta-analyses report a 94% short-term remission rate, with favorable outcomes across different Chicago classification subtypes (7). Most existing studies recruit participants with disease durations shorter than 10 years, and high-quality evidence supporting patients with decades-long esophageal fibrosis remains scarce (11, 12). Long-standing luminal obstruction drives profound anatomical remodeling of the esophagus, presenting elongated spastic segments and stiffened muscular layers. Balloon dilatation often fails to deliver sufficient sphincter release, while laparoscopic Heller myotomy is constrained by limited incision length, jointly elevating recurrence risks (13, 14). It should be emphasized that laparoscopic Heller myotomy, especially extended Heller myotomy, can be individually tailored according to disease characteristics and also achieves satisfactory clinical outcomes (15). POEM allows flexible customization of submucosal tunnel length and myotomy range according to lesion distribution. A 12-cm submucosal tunnel was constructed in this patient, facilitating full transection of spastic circular muscles without perioperative adverse events. Dysphagia resolved completely the day following surgery, accompanied by an Eckardt score of 0 and 4-kg weight recovery at the 6-month follow-up. These results validate reliable intraoperative safety and pronounced short-term symptom relief from POEM for patients with chronically remodeled esophagus from decades of untreated achalasia. Nevertheless, our follow-up period only extended six months, and long-term surveillance is required to track delayed postoperative regurgitation and symptom recurrence.

Several clear limitations characterize this investigation. Chicago subtyping could not be performed without HRM equipment, prohibiting analysis of subtype-specific differences in POEM therapeutic response. The brief follow-up window prevents evaluation of long-term prognostic endpoints. As a single-case report, its findings cannot be universally extrapolated to broader achalasia cohorts. Treatment efficacy was assessed exclusively through subjective metrics including Eckardt scoring and body mass variation. No objective functional measurements, such as esophageal emptying rate or residual lower esophageal sphincter pressure, were obtained to validate clinical recovery. We also lacked postoperative EGD and pH monitoring data. Reflux was evaluated exclusively through patient self-report, leaving subclinical reflux potentially unrecognized. Future multicenter, large-scale longitudinal cohort trials are warranted to standardize diagnostic and therapeutic pathways for HRM-deficient institutions and further define long-term clinical benefits of POEM in patients with ultra-longstanding achalasia.

## Conclusion

Achalasia is frequently misidentified as gastroesophageal reflux disease. Temporary symptom relief induced by proton pump inhibitors may lead to decades-long diagnostic delays, and persistent disease progression may cause irreversible esophageal damage and malnutrition in some patients. For patients with persistent unexplained dysphagia, repeated esophagogastroduodenoscopy should be avoided if previous endoscopies yield non-diagnostic findings. Alternative diagnostic tools including barium radiography should be considered promptly to prevent prolonged diagnostic delay. For primary care settings lacking routine access to high-resolution manometry, a non-invasive definitive diagnosis can be established by integrating typical clinical history, chest computed tomography, EGD and the pathognomonic bird-beak sign on barium radiography, forming an affordable and practical diagnostic framework. Peroral endoscopic myotomy is not constrained by achalasia subtypes and enables customized release of extensive spastic muscle segments; it maintains favorable safety and efficacy even for severely affected patients with over 20 years of disease and structural esophageal remodeling. This study reminds clinicians to abandon stereotyped empirical acid-suppressive treatment and build tiered differential diagnostic workflows for dysphagia, offering practical references for standardized management of long-duration achalasia in resource-limited medical centers.

## Data Availability

The original contributions presented in the study are included in the article/Supplementary Material, further inquiries can be directed to the corresponding author.
